# Efficacy of Selective Laser Trabeculoplasty in Medically Uncontrolled Glaucoma

**DOI:** 10.1155/2013/975281

**Published:** 2013-01-08

**Authors:** Nihat Sayin, Zeynep Alkin, Abdullah Ozkaya, Abdulvahit Demir, Ahmet Taylan Yazici, Ercument Bozkurt, Ahmet Demirok

**Affiliations:** ^1^Zonguldak Devrek Government Hospital, Devrek, Zonguldak, Turkey; ^2^Beyoglu Eye Research and Education Hospital, Bereketzade Camii Sok No. 2 Beyoglu, Istanbul, Turkey; ^3^Istanbul Medeniyet University, D-100 Karayolu Merdivenkoy Mevkii No. 6 Kadikoy, Istanbul, Turkey

## Abstract

*Purpose*. To investigate the efficacy and safety of 360° selective laser trabeculoplasty (SLT) on medically uncontrolled open-angle glaucoma (OAG) and to evaluate the effects of antiglaucomatous medications on the results of therapy. *Materials and Methods*. The medical records of 62 eyes of 51 patients with OAG, which did not reach the targeted intraocular pressure (IOP) with maximum antiglaucomatous medical therapy, were retrospectively reviewed. *Results*. A statistically significant decrease was observed in the mean baseline IOP at 1, 3, 6, and 12 months of followup (*P* < 0.01). The success rate was 64.5% in all of the patients. The success rates did not vary significantly by taking 1, 2, 3, or 4 medications with the rates of 63.6%, 71.4%, 64.2%, and 58.3% (*P* = 0.06). The success rate of eyes on medication more or less than 6 months was 62.5% or 66.7%, respectively (*P* = 0.3). There was a positive correlation between mean baseline IOP and mean reduction in IOP from baseline (*P* < 0.001, *r* = 0.8). *Conclusion*. Application of 360° of SLT provided an effective and safe IOP reduction in medically uncontrolled OAG. Baseline IOP was found to be the most important factor in the efficacy of therapy.

## 1. Introduction

Several studies have corroborated the efficacy and safety of selective laser trabeculoplasty (SLT) since its first description in 1995 by Latina and Park [1]. Selective laser trabeculoplasty, which uses a 532 nm frequency-doubled, Q-switched Nd:YAG laser, is thought to selectively stimulate the pigmented trabecular meshwork cells and thus facilitate improved aqueous outflow in open-angle glaucoma (OAG) [2]. 

A synthesis of previous data suggests that SLT is effective at every stage of treatment for OAG. Selective laser therapy can be used as a first-line therapy, alternative to medical therapy, or as an adjunctive therapy to topical glaucoma drops [3, 4]. Although success rates and levels of reported IOP reduction vary between studies, a great number of studies show that it is a safe procedure with low complication rates [5–7]. This heterogeneity could be explained by differences in the samples, outcomes, and treatment protocols such as power, spot numbers, and the degree of angle treated.

The aims of this study were to investigate the safety and efficacy of 360° SLT application in patients with OAG who were not well controlled with maximal medical therapy and also to evaluate the factors that could influence the results including the number and type of antiglaucomatous medications, duration of medical therapy, and baseline IOP. 

## 2. Materials and Methods

This was a retrospective chart review of patients treated with SLT from April 2009 to February 2011. Approval for data collection and analysis was obtained from the ethics committee of the hospital, and all patients provided informed consent. The methodology of the study was designed in accordance with the tenets of the Helsinki Declaration.

Inclusion criteria were OAG (primary open-angle, pseudoexfoliation glaucoma) patients who were older than 18 years and did not reach the target intraocular pressure (IOP) with maximal medical therapy. Exclusion criteria were congenital glaucoma, any type of angle closure glaucoma, advanced-stage glaucoma, eyes with previous laser or surgical glaucoma applications, and eyes with previous anterior segment surgery within the past 6 months. Patients who could not be followed for at least 12 months were also excluded. 

Data recorded from each patient included age, sex, type of glaucoma, lens status, antiglaucoma medications, duration of medical therapy, systemic diseases, and the SLT protocol (number of spots and laser power settings). Baseline data were obtained for each patient before the treatment, which included the best corrected visual acuity (BCVA) with ETDRS chart, slit lamp biomicroscopy, Goldmann applanation tonometry, gonioscopy, mydriatic fundoscopy, and Humphrey 30-2 computerized perimetry. Follow-ups were scheduled to take place at 1, 3, 6, and 12 months after SLT. At each follow-up visit, an ophthalmic examination, which included visual acuity measurement, slit lamp biomicroscopy, and Goldmann applanation tonometry, was performed. Additionally, IOP in the treated eye was assessed and recorded 1 hour following SLT. If there was an IOP rise greater than 5 mm Hg from baseline IOP, the elevation of the IOP was treated with appropriate antiglaucoma medications. 

Immediately before the laser procedure, a single application of proparacaine hydrochloride 0.5% was instilled into the eye. All patients were treated by the same physician (N. S). The 360° SLT treatment protocol was performed the entire meshwork with the SLT Laserex Tango laser system (Laserex Tango, Ellex Medical, Australia) in all cases. The energy level ranged from 0.6 to 1.0 mJ. The laser energy was initially set at 0.6 mJ and a single laser pulse was delivered at the 12 o'clock position. If a cavitation bubble appeared, the laser energy was reduced by 0.1 mJ increments until no bubble formation was observed; treatment was then continued at this energy level. If no cavitation bubble was observed, the pulse energy was increased by increments of 0.1 mJ until bubble formation and then decreased as described above. Apraclonidine was instilled once after SLT. All patients continued with the same medical treatment after SLT. Postoperatively, the patients were prescribed fluorometholone 0.1% eye drops 4 times a day for 7 days. Any symptoms of ocular complications were recorded.

### 2.1. Statistical Analysis

Success was defined as ≥20% reduction in IOP from baseline at 12 months after treatment with no additional antiglaucomatous interventions. Visual acuity was converted to the logarithm of the minimum angle of resolution (log MAR) for statistical analysis. A repeated measures analysis of variance was performed to analyze the changes in BCVA and IOP from baseline to 1, 3, 6, and 12 months of therapy. When the *P* value from the analysis of variance was significant, pairwise comparisons were used to compare those variables at different time points. To identify the effect of baseline IOP on the reduction in IOP, we performed the Pearson correlation analysis. For analysis of success rates in association with type, number, and duration of antiglaucoma medications, a nonparametric Fisher's exact test was used. The statistical evaluation was performed using SPSS (Version 16.0, SPSS Inc., Chicago, IL, USA). A *P* value of less than 0.05 was considered to be statistically significant.

## 3. Results

Sixty-two eyes of 51 patients were included in the study. The baseline characteristics of the patients are listed in Table 1.

### 3.1. Efficacy of the Therapy

**Figure 1:**
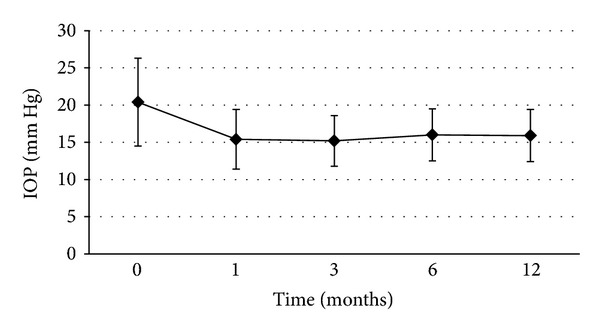
The course of the mean IOP during the follow-up period.

After SLT, the mean baseline IOP decreased from 20.4 ± 5.9 mm Hg to 15.4 ± 4 mm Hg, 15.2 ± 3.4 mm Hg, 16 ± 3.5 mm Hg, and 15.9 ± 3.5 mm Hg at 1, 3, 6, and 12 months of therapy (*P* < 0.01 for each), respectively, as shown in Figure 1. Out of the eyes studied, 40 (64.5%) achieved an IOP reduction of ≥20% from baseline at 12 months. 

The mean baseline IOP was strongly associated with the mean reduction in pressure after SLT at 12 months (*P* < 0.001, *r* = 0.8). Twenty-two patients (35.5%) were on only one medication (PGA) prior to SLT treatment, 14 (22.5%) on 2 medications (*β*-blocker, CA), 14 (22.5%) on 3 medications (PGA, *β*-blocker, and CA), and 12 (19.5%) on 4 medications (PGA, *β*-blocker, CA, and *α*2-agonist). The success rates did not vary significantly by taking either 1 or more medications with the rates of 63.6% of eyes on 1 medication, 71.4% of eyes on 2 medications, 64.2% of eyes on 3 medications, and 58.3% of eyes on 4 medications (*P* = 0.06). The success rate of eyes on medication for more than 6 months (mean 28.2 months) was 62.5% and compared to the success rate of eyes on medication for less than 6 months (mean 2.1 months) which was 66.7%. No significant difference was found at month 12 (*P* = 0.3). 

The mean BCVA was 0.3 ± 0.2 log MAR at baseline and 0.3 ± 0.4 log MAR at month 12; no significant change was observed in the mean BCVA (*P* = 0.6). 

There were no significant complications during the follow-up period. There was a minimal inflammatory reaction (1 + cells) in 9 eyes (14.5%) at 1 hour, and none of the patients had a persistent inflammatory reaction. A pressure spike, defined as an IOP elevation >5 mm Hg above pretreatment IOP, at 1 hour after laser therapy was found in 13 eyes (20.9%). Ocular discomfort, pain, and conjunctival redness were detected in 13 eyes (20.9%).

## 4. Discussion

Previous reports showed that SLT is a viable option as a primary or adjuvant treatment for patients with ocular hypertension, POAG, and PEG [3, 8, 9]. Some authors reported that SLT may not be as effective when used in conjunction with PGAs owing to the potential of competing pathways [10]. In contrast, Singh et al. [11] suggested that the IOP-lowering efficacy of SLT is not influenced by the use of topical PGAs. Our results showed that success rates were not different between PGA users and *β*-Blocker-CA combination users. 

Kano et al. [12] found that the success was correlated with the preoperative IOP, but the other variables, such as age, gender, prior ALT, and angle pigmentation, were not significant factors. A conflicting result from Sweden suggested that increased angle pigmentation and degree of exfoliation significantly correlate with higher success rates [13]. Mao et al. [14] speculated that prolonged exposure of TM to elevated IOP may negatively affect SLT results by reducing the impact of laser treatment. In this study, SLT efficacy was positively associated with higher IOP levels before SLT treatment. 

Selective laser trabeculoplasty is still used by many clinicians as an adjunctive treatment, rather than a primary therapy. Therefore, its performance with adjunctive medical therapy is critical in understanding its role in the glaucoma treatment algorithm. Hence, we evaluated the number, and duration of medications before SLT. In this study, we compared the SLT success in eyes taking 1 or more antiglaucomatous medications in patients with OAG. We found no significant difference in success rates between the patients who were on 1 medication and 2 or more medications. Our study also showed that the duration of antiglaucomatous medications had no effect on success rates at 12 months of therapy. However, in Song's study, the failure rate (73%) was highest among the eyes on 4 medications, and eyes on no medication had the lowest failure rate (50%) [15]. In the same study, the groups of patients who were on a different number of medications consisted of those who received a different class of drops from those in our study. 

In the published studies to date, the success rate of SLT varies from 40% to 84% [3, 12, 13, 15–20]. Nagar et al. [21] reported very encouraging results in success rates of 360°SLT, with 80% of eyes achieving a 20% or more IOP reduction at 12 months of therapy. The effect of the SLT treatment in the present study was less potent than Nagar's result indicated. This could be because the patients in the present study probably had lower baseline IOP than those in Nagar's study. 

One hour after SLT, 29% of the patients had an IOP spike of more than 5 mm Hg in our study. The rate of IOP spike from a previous report [21], with the rate of 27%, was comparable with our results. 

This study was limited by the small number of patients and by its retrospective study design. In summary, SLT treatment is an effective laser procedure for lowering IOP in OAG. Also, SLT success rates are not influenced by type, number, and duration of topical antiglaucomatous medications. Further long-term prospective studies on a larger subject population may likely change our results.

## Figures and Tables

**Table 1:** Baseline characteristics of the patients included in the study.

Characteristics	
Age ± SD	68.7 ± 9.9
Gender, female (%)	48%
Lens status, phakic (%)	74.2%
Baseline IOP (mm Hg) ± SD	20.4 ± 5.9
Baseline BCVA (log MAR) ± SD	0.3 ± 0.2

Systemic disease (%)	
Diabetes mellitus	7.8%
Arterial hypertension	25.4%

Type of glaucoma (%)	
POAG	54.8%
PEG	45.2%

TM pigmentation ± SD	2.3 ± 0.5

Procedure parameters	
Number of laser spots ± SD	102.4 ± 21.7
Total energy ± SD	80.7 ± 16

SD: standard deviation; BCVA: best corrected visual acuity; POAG: primary open-angle glaucoma; PEG: pseudoexfoliative glaucoma; TM: trabecular meshwork.
